# The Extent of Genome Flux and Its Role in the Differentiation of Bacterial Lineages

**DOI:** 10.1093/gbe/evu123

**Published:** 2014-06-12

**Authors:** Reuben W. Nowell, Sarah Green, Bridget E. Laue, Paul M. Sharp

**Affiliations:** ^1^Institute of Evolutionary Biology, University of Edinburgh, United Kingdom; ^2^Forest Research, Centre for Ecosystems, Society and Biosecurity, Roslin, Midlothian, United Kingdom; ^3^Centre for Immunity, Infection and Evolution, University of Edinburgh, United Kingdom

**Keywords:** bacterial genome fluctuation, horizontal gene transfer, adaptation, speciation, *Pseudomonas syringae*

## Abstract

Horizontal gene transfer (HGT) and gene loss are key processes in bacterial evolution. However, the role of gene gain and loss in the emergence and maintenance of ecologically differentiated bacterial populations remains an open question. Here, we use whole-genome sequence data to quantify gene gain and loss for 27 lineages of the plant-associated bacterium *Pseudomonas syringae*. We apply an extensive error-control procedure that accounts for errors in draft genome data and greatly improves the accuracy of patterns of gene occurrence among these genomes. We demonstrate a history of extensive genome fluctuation for this species and show that individual lineages could have acquired thousands of genes in the same period in which a 1% amino acid divergence accrues in the core genome. Elucidating the dynamics of genome fluctuation reveals the rapid turnover of gained genes, such that the majority of recently gained genes are quickly lost. Despite high observed rates of fluctuation, a phylogeny inferred from patterns of gene occurrence is similar to a phylogeny based on amino acid replacements within the core genome. Furthermore, the core genome phylogeny suggests that *P. syringae* should be considered a number of distinct species, with levels of divergence at least equivalent to those between recognized bacterial species. Gained genes are transferred from a variety of sources, reflecting the depth and diversity of the potential gene pool available via HGT. Overall, our results provide further insights into the evolutionary dynamics of genome fluctuation and implicate HGT as a major factor contributing to the diversification of *P. syringae* lineages.

## Introduction

Bacterial genomes are labile entities, fluctuating in both size and content through time (Ochman et al. 2005; Hao and Golding 2006; Touchon et al. 2009). Genome fluctuation is mediated by the counteracting processes of gene gain and gene loss. The addition of novel genes expands the functional diversity of the recipient genome, whereas genes whose presence is no longer required for survival can be deleted. The addition of new genes occurs primarily through horizontal (lateral) gene transfer (HGT), which can drive both phenotypic innovation and subsequent adaptation when the acquired genes confer new traits that allow diversification of that lineage into a novel environment (Ochman et al. 2000; Boucher et al. 2003; Lerat et al. 2005; Treangen and Rocha 2011). However, the specific contribution of HGT toward other evolutionary processes, such as genomic differentiation and the formation of ecologically defined population structure, from which speciation may result, remains an open question (Lawrence 2002; Ochman et al. 2005; Polz et al. 2013).

High rates of gene exchange due to HGT can lead to extensive gene repertoire differences among otherwise closely related lineages and can result in genes within the same genome having conflicting evolutionary histories (Feil et al. 2001; Gogarten et al. 2002; Ochman et al. 2005; Shapiro et al. 2012). Thus, HGT is predicted to obfuscate patterns of vertically inherited differences between lineages and should undermine the definition of bacterial “species” as nonrecombining taxonomic units (Gogarten et al. 2002; Doolittle and Papke 2006; Doolittle and Zhaxybayeva 2009). However, despite overwhelming evidence of extensive HGT at virtually all phylogenetic levels, bacterial lineages generally do behave as distinct entities that often appear to have well-defined boundaries between taxonomic groups (Daubin et al. 2003; Ochman et al. 2005; Hanage et al. 2006; Caro-Quintero and Konstantinidis 2011). These apparently conflicting observations have led to the proposition that the majority of HGT is transient and therefore does not impact the evolutionary trajectories of bacterial lineages (Hao and Golding 2006; Kuo and Ochman 2009). Furthermore, recent work has suggested that HGT may play an important role in the evolution of population structure within bacterial communities and may facilitate the speciation process (Ochman et al. 2005; Polz et al. 2013).

HGT may bring about divergence and differentiation if the diversifying effect of HGT is able to subdivide a population to the extent that speciation can occur (Lawrence and Ochman 1998; Lawrence 1999, 2002; Ochman et al. 2005; Barraclough et al. 2012; Polz et al. 2013). Under this model, variation in the functions of acquired genes results in the differential adaptation of subpopulations of bacteria into correspondingly different ecological habitats or niches, thereby creating a physical barrier to continued exchange and allowing sequence divergence to accrue to the extent that homologous recombination is suppressed. Subsequent gene loss can enhance differentiation between lineages by limiting niche overlap between incipient species through the restriction of metabolic diversity of populations of bacteria (Lawrence 1999). The innovative and diversifying effects of HGT are well documented, and as such, the exposure to a potentially huge pool of novel genes via HGT could be the mechanism that drives the early stages of this process. Thus, the forces of genome fluctuation, HGT, and adaptation are inexorably intertwined and can, under the right conditions, act in concert to promote both genomic differentiation and ecological diversification, from which speciation can result.

Here, we examine the interplay between these evolutionary processes using the *Pseudomonas syringae* species complex as a model system. *Pseudomonas syringae* is a remarkably diverse species found almost globally in a multitude of different environments (Hirano and Upper 1990; Sarkar and Guttman 2004). Although best known as a plant pathogen, it can also exist as a plant commensal or a free living organism capable of thriving in nonagricultural habitats (Sarkar and Guttman 2004; Morris et al. 2008, 2010). The species complex comprises over 50 pathovars (pathological variants) delimited into a number of phylogroups based on sequence divergence (Sarkar and Guttman 2004; Hwang et al. 2005; Parkinson et al. 2010). Although as a species *P. syringae* has a broad host range, individual strains are generally confined to a limited set of potential host species (Sarkar et al. 2006; Lindeberg et al. 2009). Strains injected into a nonhost plant will either simply fail to grow or will elicit an immune response from the plant that halts the progression of disease symptoms (Morris et al. 2000; Hunter and Taylor 2006; Sarkar et al. 2006). Thus, individual strains have specific genetic repertoires that promote pathogenicity only when expressed in a particular environment, that is, that of a compatible host species (Lin and Martin 2005; Sarkar et al. 2006).

The *P. syringae* species complex offers a unique opportunity to study the contribution of HGT to the early stages of differentiation between lineages for a number of reasons. First, the radiation of *P. syringae* pathovars onto multiple different host species combined with the high degree of host specificity of individual pathovars allows for an investigation into the role that HGT may have in the partitioning of the *P. syringae* species complex into different ecological niches, where in this case each host species can be viewed as a distinct niche environment to which its *P. syringae* pathovar is specifically adapted. Second, the *P. syringae* species complex is an example of a group of bacteria in the early- to midstages of differentiation, with a number of clades (i.e., phylogroups) with a level of sequence divergence in core genes comparable with that of other “young” bacterial species. Finally, there exists a good representation of whole-genome sequence data across at least three major phylogroups within the complex, allowing for the accurate elucidation of genome content variation across closely related but phenotypically distinct lineages.

We utilize these data to quantify the extent of gene gain and loss within a phylogenetic framework for 27 lineages of the *P. syringae* species complex. Using available whole-genome sequence data and a suite of tools developed to limit the confounding effects of errors associated with draft genomes, we decompose the *P. syringae* pan genome into its constituent core and flexible components and apply various phylogenomic methods to infer the evolutionary histories of both these entities. We reconstruct the most likely history of gene gain and loss within the framework of the evolutionary history of the core genome and correlate the rate of gain and loss with phylogenetic depth to provide insights into the dynamics of genome fluctuation through time. We then classify gained genes based on both putative function and potential source to elucidate the functional diversity and the taxonomic breadth of donor species of gained genes. We use these results to ask if the divergence via differential adaptation model described above can explain the observed radiation of *P. syringae* pathovars onto different host species. If this were the case, we would expect to see the acquisition of genes along phylogenetic branches that correspond to host adaptation events. Of particular importance in this process are genes of the type III secretion system (T3SS). This “molecular syringe” is a fundamental component of the *P. syringae* armory that knocks down innate plant immune defenses via the injection of type III secretion effector proteins (T3SEs) into host cells, allowing for infection (Lindeberg et al. 2009, 2012). Thus, the repertoire of T3SEs possessed by a given pathovar is a key determinant of host specificity, and the gain of T3SEs may play an important role in the diversification of *P. syringae* lineages onto novel host species (Sawada et al. 1999; Ma et al. 2006; Lindeberg et al. 2009; Baltrus et al. 2011; O'Brien et al. 2012).

## Materials and Methods

### Genomic Data

All available *P. syringae* genome sequences (as of May 4, 2012) were downloaded from National Center for Biotechnology Information (NCBI) GenBank, including multiple strains of the same pathovar (but excluding multiple editions of the same strain). This yielded 27 strains of 18 pathovars from across the *P. syringae* species complex. Summary information for all genomes included in this analysis is given in table 1 and supplementary table S1, Supplementary Material online. All protein sequences used in subsequent analyses were based on the published annotations for these genome sequences, also retrieved from NCBI GenBank.

**Table 1:** Summary Information for 27 *Pseudomonas syringae* Pathovars Used in this Study

Pathovar	Strain	Tag^a^	Species of Isolation	Contigs^b^	Proteins^c^
*aceris*	M302273PT	acer	*Acer* sp. (maple)	1,179	6,185
*actinidiae*	M302091	actn	*Actinidia delicosa* (kiwi fruit)	941	5,626
*aesculi*	2250	aes2	*Aesculus hippocastanum* (European horse chestnut)	776	5,619
*aesculi*	NCPPB 3681	aesN	*Aesculus indica* (Indian horse chestnut)	841	5,649
*aptata*	DSM 50252	apta	*Beta vulgaris* (sugar beet)	3,776	6,368
“*cit7*”	Cit7	cit7	*Citrus sinensis* (navel orange)	2,655	7,145
*glycinea*	B076	glyB	*Glycine max* (soybean)	104	5,580
*glycinea*	race 4	gly4	*G. max* (soybean)	108	5,207
*japonica*	M301072PT	japa	*Hordeum vulgare* (barley)	4,661	8,796
*lachrymans*	M301315	lacM	*Cucumis sativus* (cucumber)	791	6,691
*lachrymans*	M302278PT	lacP	*C. sativus* (cucumber)	798	5,636
*maculicola*	ES4326	Pcan	*Raphanus sativus* (radish)	878	6,101
*mori*	M301020	mori	*Morus alba* (white mulberry)	3,414	7,544
*morsprunorum*	M302280PT	mrsp	*Prunus domesticus* (European plum)	969	5,837
*oryzae*	1_6	oryz	*Oryza sativa* (rice plant)	2,855	7,837
*phaseolicola*	1448A	phas	*Phaseolus vulgaris* (common bean)	3	5,172
*pisi*	1704B	pisi	*Pisum sativum* (pea)	5,099	9,160
*savastanoi*	NCPPB 3335	sava	*Olea europaea* (olive tree)	403	4,898
*syringae*	B728a	syrB	*P. vulgaris* (common bean)	1	5,089
*syringae*	FF5	syrF	*Pyrus calleryana* (ornamental pear)	4,578	8,445
*syringae*	642	syr6	Unspecified	296	5,274
*tabaci*	ATCC 11528	taba	*Nicotiana tabacum* (tobacco plant)	1,405	6,467
*tomato*	DC3000	tomD	*Solanum lycopersicum* (tomato)	3	5,619
*tomato*	K40	tomK	*S. lycopersicum* (tomato)	582	5,853
*tomato*	Max13	tomM	*S. lycopersicum* (tomato)	349	5,749
*tomato*	NCPPB 1108	tomN	*S. lycopersicum* (tomato)	304	5,619
*tomato*	T1	tomT	*S. lycopersicum* (tomato)	122	5,702

Note.—More information, including genome references, can be found in supplementary table S1, Supplementary Material online.

^a^Four-letter identifier used in this study.

^b^The number of contiguous sequences given for each genome.

**^c^**The number of proteins given for each genome.

### Assignment of Orthology

Orthologous relationships among proteins were determined using OrthoMCL version 2.0 (Li et al. 2003). OrthoMCL uses Basic Local Alignment Search Tool (BLAST) followed by Markov clustering (MCL) to group proteins into putative orthologous groups (OGs). A minimum length cutoff of 50 residues was chosen as many sequences less than 50 were found to be protein fragments (see below). The all-versus-all BLASTp was performed with an *E* value ≤ 1 × 10^−5^, and the inflation index, which controls the threshold at which the MCL algorithm defines distinct OGs, was set at 1.5 (Li et al. 2003; Lefébure and Stanhope 2007; Lefébure et al. 2010).

### Correction of Errors Associated with Draft Genome Sequence Data

The resultant list of OGs was subjected to a series of quality control procedures to minimize the confounding effects of errors such as misannotations and (in particular) the presence of protein fragments. Protein fragments are caused by the premature truncation of a protein-coding sequence (CDS) in the genome by the termination of a contig in the middle of an open reading frame. When a CDS spans a break between two adjacent contigs (i.e., a gap in a draft genome), it is possible to get both the 5'- and 3'-portions of the split gene being annotated as distinct entities (with unique protein accession numbers) within the proteome of that genome.

The presence of these fragments causes error in the inference of orthology via two primary mechanisms. First, fragments can cluster to form an OG that is distinct from the group containing their full-length true co-orthologs. This introduces an additional, spurious OG to the analysis and can lead to an overestimate in the number of gains and/or losses. Second, protein translations of both the 5'- and 3'-fractions of a split CDS may cluster within the same OG, giving rise to multiple proteins from the same genome within a given OG (i.e., apparent paralogs). This can lead to an underestimate of the core genome size when only genes in single copy per genome are counted. In addition, fragments that do not cluster in any OG can overestimate the number of lineage-specific proteins per pathovar. To account for these issues, a series of filters (written in Perl) were developed and are briefly discussed below.

To correct for entirely spurious OGs, the consensus sequences of all 2,358 2-member OGs (“twosomes”) were used to search a database consisting of the consensus sequences of all 10,320 OGs inferred by OrthoMCL. Alignments were constructed using T-Coffee version 8.99 with default parameters (Notredame et al. 2000), and BLASTp was run at *E* value ≤ 1 × 10^−5^. Query sequences with a significant hit (≥80% amino acid identity over ≥80% query length) to another alignment were classified as spurious and the corresponding twosome OG was removed from the analysis. The process was repeated iteratively over increasing OG size classes (threesomes, foursomes, etc.) until the method inferred an error rate of <0.05% in the OG data set (at tensomes).

To correct for OGs containing multiple fragments of the same CDS, the alignments of all groups that had co-orthologous proteins from the same genome (i.e., apparent paralogs) were subjected to further investigation. For alignments that had an average pairwise amino acid identity of ≥80% and that showed evidence that the two paralogs were in fact fragments, only the longest fragment of any split protein was retained in the alignment. A further 499 OGs were removed from all subsequent analyses due to poor alignment and/or ambiguous orthology.

Any sequences with homology to any known insertion sequence-related proteins were removed from the data set by querying the 59 known *P. syringae* IS elements (Bardaji et al. 2011), downloaded from “IS Finder” at www-is.biotoul.fr/ (last accessed April 24, 2013), against the combined input proteomic data set (BLASTp, *E* value ≤ 1 × 10^−10^). Any OG that contained a sequence with ≥70% pairwise amino acid identity was removed from subsequent analyses.

### Construction of Core and Lineage-Specific Gene Sets

The resultant lists of OGs from the OrthoMCL analysis were decomposed into putative pan-, core-, and lineage-specific gene sets using a further set of custom Perl scripts. Only core genes with no paralogs (i.e., 1-to-1 orthologs) were included in the core gene set. Because the exclusion of a single pathovar from an “almost-core” OG necessarily excludes that gene from the core set as defined above, all 969 OGs that had a member protein in 26 of the 27 genomes were further scrutinized to ensure the nonexistence of an ortholog in the nonparticipating genome, using tBLASTn to account for the possibility of annotation errors. Briefly, this filter queried each nonparticipating genome with the corresponding ortholog from the pv. *phaseolicola* str. 1448A (*phas*) genome, using tBLASTn (*E* value ≤ 1 × 10^−5^). All hits that had an 80% or greater amino acid identity over 80% or more of the query length were considered homologous. When pv. *phaseolicola* itself was the nonparticipating genome, then the corresponding ortholog from pv. *tomato* str. DC3000 (*tomD*) was used.

All sequences that did not cluster to form an OG were compiled into an initial lineage-specific gene set. The presence of protein fragments (discussed above) is known to erroneously inflate this gene set (Lefébure and Stanhope 2007; Lefébure et al. 2010; Richards et al. 2011). Thus, a protein was considered lineage specific only if it returned no significant hits (other than to self) when queried against the combined genomic data set, using tBLASTn (*E* value ≤ 1 × 10^−10^).

A binary matrix of gene presence or absence across all OGs and pathovars was used to plot gene accumulation curves with a custom R script; for each comparison, genome input order was randomly permuted 100 times to acquire sample distributions.

### Core Genome Phylogenetics

The genome-wide average evolutionary history for these *P. syringae* strains was inferred using a core genome concatenation approach. For each core OG, protein sequences were aligned using T-Coffee. All sites that contained a gap in any sequence were excluded. The presence of truncated sequences in many alignments meant that the exclusion of gap sites sometimes ruled out the entire alignment (i.e., when all alignment columns had at least one gap). Alignments with low identity (<30% identical sites), short length (<30 residues), or 100% identity were not included in the concatenation. The remaining 2,140 alignments were concatenated, and a maximum-likelihood tree was built with PhyML version 3.0 (Guindon and Gascuel 2003; Guindon et al. 2010) using the Le and Gascuel (2008) model of amino acid substitution with four gamma-distributed rate categories (LG + G) and a combination of nearest-neighbor interchange (NNI) and subtree-prune-regraft (SPR) methods of tree topology search; 100 bootstrap replicates were performed to test topological robustness. A maximum-likelihood phylogeny based on over 1 million core nucleotide sites was also constructed in PhyML, using a GTR + G model with four rate categories, NNI + SPR branch swapping, and 100 bootstrap replicates.

Maximum-likelihood phylogenies for all 2,140 individual core alignments were constructed at both the DNA and protein level, using the same parameters as above. Topological concordance among the set of phylogenies was examined by using the program “consense” from the Phylip package (Felsenstein 1981) to build a consensus tree (extended majority rule).

### Genome Content Analysis

To estimate a phylogeny based on genome similarity in terms of gene content, a distance matrix was constructed where the distance measured between two taxa is inversely proportional to the number of genes shared between them (Snel et al. 1999; Kettler et al. 2007; Georgiades et al. 2011). If gain and loss are assumed to affect the flexible genome only, the distance between genome A and B (*d*_AB_) can be calculated as follows: *d*_AB_ = 1−((*S*_AB_* − C*)/(*T − C*)), where *S*_AB_ is the number of shared genes, *C* is the core genome size, and *T* is the total number of proteins in the smaller of the two genomes. To correct for unobservable events along deeper branches in the phylogeny, a Poisson correction procedure was applied as follows: *d'*_AB_ = −ln(1 − *d*_AB_). A phylogeny was then inferred using the neighbor-joining method (Saitou and Nei 1987) with 1,000 bootstrap replicates sampling columns of the presence/absence matrix, and recording the consensus support over the bootstrap data sets at each node using consense.

Levels of synonymous (*K*_S_) and nonsynonymous (*K*_A_) divergence between all pairs of taxa within and between the major clades in the core-genome phylogeny were estimated for the same 2,140 core nucleotide alignments (over 1 million sites), using the method of Li (1993), implemented through the R package “SeqinR” (Charif and Lobry 2007).

### Reconstruction of Gene Gain and Loss

The history of gene gain and loss was analyzed using a stochastic mapping method implemented through the GLOOME server (http://gloome.tau.ac.il/, last accessed October 23, 2013) (Cohen et al. 2010; Cohen and Pupko 2010, 2011). The GLOOME algorithm takes as input the phyletic matrix of gene-family presence and absence and a reference phylogenetic tree, and infers probabilities and expectations for all gain and loss events in a per site per branch manner. This method may be particularly suitable for the analysis of genomic data sets that are split into numerous contigs (i.e., draft genomes), because it does not require gene synteny information. In addition, other methods that reconcile differences between a gene tree and a species tree to infer duplication, transfer, and loss events often require fully binary trees—a condition that is often not met in comparative genomic analyses of closely related taxa where there may be poor phylogenetic resolution at the level of the individual gene. Stochastic mapping allows for the total number of gains and losses along a given phylogenetic branch to be calculated, as well as the subsequent extraction of specific gain events that have a high associated probability. Probabilistic models of gene gain and loss are superior to parsimony-based approaches because the rate of gain and loss is allowed to vary among OGs, and both topology and branch lengths are considered in the estimation of associated probabilities (Cohen and Pupko 2010). The core genome phylogeny was used as the reference tree, and the total number of gains for each branch was calculated as the probability of gain for each gene, summed across all genes and rounded to the nearest whole number. The procedure was repeated for losses. A branch-specific rate of gene gain/loss was also calculated as the ratio of the number of events (gains or losses) to the length of that branch, equivalent to the number of events per 1% amino acid sequence change of the core genome.

Using the stochastic mapping results, genes with a gain probability of ≥0.8 were extracted on a branch-by-branch basis for functional categorization. A cutoff of 0.8 was chosen because this was the lowest observed probability value with which lineage-specific genes were inferred to have been gained on the terminal branches of the tree, that is, a more conservative cutoff value may have excluded some lineage-specific genes from the analysis. For each branch, a reference sequence from each gained OG was chosen randomly and functionally annotated using Blast2GO version. 2.5.0 under default settings (Conesa et al. 2005). Gained sequences were initially classified into one of three (mutually exclusive) basic categories: “functional protein” if the sequence was successfully annotated within Blast2GO, “hypothetical protein” if no function was ascribed, and “phage related” if that sequence had matches to known phage-related proteins. Those sequences that were ascribed functions were further delimited based on their associated gene ontology (GO) terms. Only branches with ≥10 gains were analyzed, and only GO categories with ≥3 sequences are shown. The functional protein category was chosen for further GO classification to ascertain the underlying functional diversity of gained sequences.

The potential source of recently transferred genes was investigated using a BLAST-based approach. Protein sequences for all genes gained along terminal branches (i.e., recent acquisitions) with a gain probability of ≥0.8 were queried against the NCBI nonredundant database using BLASTp (*E* value ≤ 1 × 10^−20^). For each query, the donor species was inferred to be the most similar match with the lowest BLAST *E* value. This approach will miss potential donor species that are not represented within the database but allows for an approximate estimation of the extent to which HGT occurs within phylogroups, between phylogroups or from other species.

To assess the extent to which effector genes of the T3SS comprise recently acquired genes, all terminally gained proteins were queried against a database of all currently described type III secretion system effector proteins (T3SEs) using BLASTp. The database was constructed using the T3SE sequence information provided at www.pseudomonas-syringae.org (last accessed August 13, 2013) and by Wang et al. (2012) and consists of >1,700 T3SS-related protein products. Matches with ≥80% identity over ≥80% of the query length were inferred to be homologous to a T3SE.

## Results

### The Core and Pan Genomes of *P. syringae*

**Fig. 1.—:**
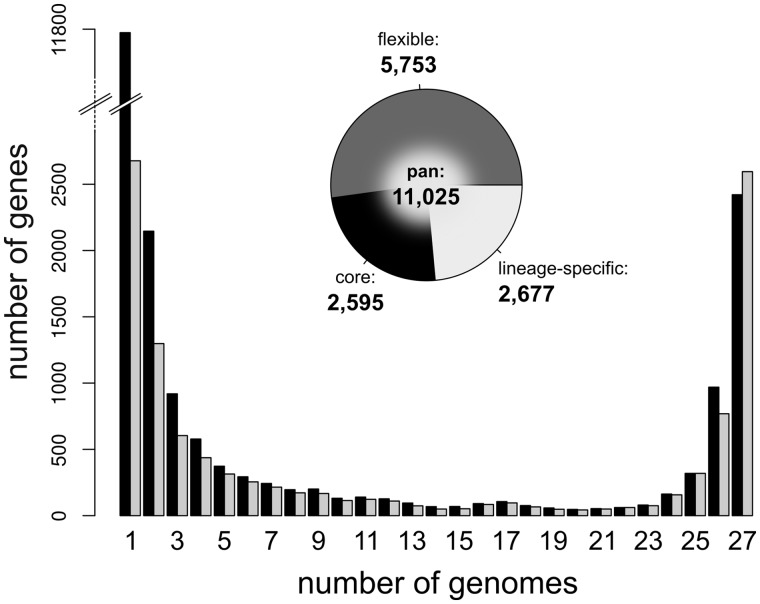
Frequency distribution of genes across genomes. Black bars represent the number of genes in each OG size category prior to the various correction procedures outlined in Materials and Methods; gray bars are corrected values. The pie chart shows the delineation of the *Pseudomonas syringae* pan genome into its constituent entities: Core (i.e., genes present in all strains), flexible (i.e., genes with a variable occurrence across strains), and lineage specific (i.e., genes unique to individual strains).

We compared the genome sequences of 27 strains of *P. syringae*. Initially, these showed a combined total of 159,748 proteins, of which 148,254 (∼93%) were clustered into a total of 10,320 inferred OGs by OrthoMCL. The relative frequency across genomes of each OG size class is shown before and after correction for errors (fig. 1). Prior to correction, 11,791 genes (∼53% of the initial estimate of the pan genome) were inferred to be lineage specific. After accounting for errors due (primarily) to the presence of protein fragments, this value was reduced by more than three-quarters to only 2,677 genes (∼12% of the initial estimate of the pan genome). The shape of the corrected distribution suggests that the majority (∼75%) of genes of the pan genome are found in either very few (≤3) or most (≥25) genomes, with a paucity of genes distributed in the mid range of the distribution.

**Fig. 2.—:**
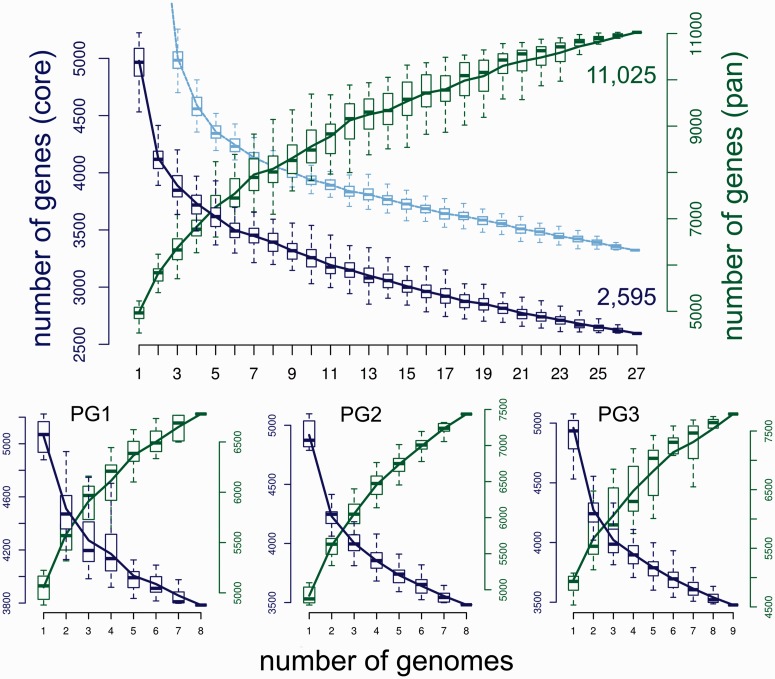
Core and pan genomes of *Pseudomonas syringae*. The upper figure shows gene accumulation curves for the core (dark blue) and pan (green) genomes for the combined data set (27 genomes). The light blue curve shows the core estimate when almost core genes (i.e., genes present in 26 genomes) are also included (comparisons of <3 genomes are not shown for this curve). The dark blue axis on the left shows the number of genes of the core genome, and the green axis on the right shows the number of genes of the pan genome (note the different scales). Boxes show the interquartile range of 100 random permutations of genome input order for the given number of genomes in comparison; the horizontal line shows the median value. The lower figure shows the equivalent results when the data are partitioned into the three major phylogroups. PG1, PG2, and PG3 represent phylogroups 1, 2, and 3, respectively.

After correction, the size of the pan genome for all 27 lineages was 11,025 genes (fig. 2, upper panel). However, the curve did not tend toward an asymptote, suggesting that a considerable amount of variation (in terms of novel genes) remains to be discovered with the addition of further genomes. The core genome of the entire *P. syringae* species complex was initially estimated to be 2,421 genes. Correction for missed core genes increased this value by 174 to 2,595 genes or approximately 52% of the average *P. syringae* genome content. Again, the shape of the curve suggests that the number of core genes will decrease further given additional genome sampling. We considered the effect of missed genes due to incomplete genome sequences by running an analysis where almost core genes (with a member in *n* − 1 genomes) were included. This increased the estimated core genome to 3,364 genes but had little effect on the shape of the accumulation curve, suggesting that the failure of the original curve to tend toward an asymptote is not due to missed genes. When analyzed individually, pan genome estimates for phylogroups 1, 2, and 3 were 6,778, 7,436, and 7,790 genes, respectively, and estimates for the core genome were 3,785, 3,481, and 3,477 genes, respectively (fig. 2, lower panel). These values are substantially lower (pan) and higher (core) when compared with the corresponding value under the appropriate number of genomes in the full analysis.

### *Pseudomonas syringae* Phylogenetics

**Fig. 3.—:**
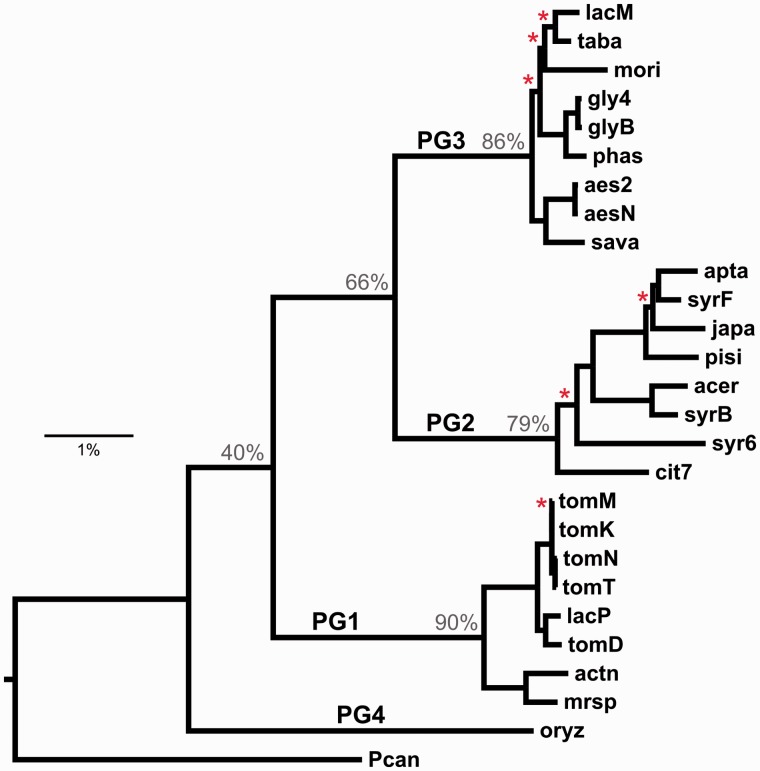
Core genome phylogeny for *Pseudomonas syringae*, derived from 2,140 concatenated protein alignments. All internal branches have 100% bootstrap support except where indicated with asterisks. Consensus support from individual gene trees is shown with gray numbers on major branches, and phylogroups PG1–PG4 are indicated. Tree is rooted with pv. *maculicola* str. ES4326 (*Pcan*). Scale bar indicates 1% amino acid replacements per site.

The core genome phylogeny for *P. syringae* was estimated from 2,140 concatenated proteins totaling 351,468 aligned residues (fig. 3). Pathovar *maculicola* str. ES4326 (*Pcan*) was defined as the outgroup to root the core genome phylogeny, because it has been reported that this pathovar was originally misidentified and is in fact a strain of *P. cannabina* (Baltrus et al. 2011), a closely related species outwith the *P. syringae* species complex (Parkinson et al. 2010). Bootstrap support of branching patterns was generally very high with only one partition showing less than 75% support. The phylogeny delineates the 26 ingroup lineages into four clades, in agreement with the phylogroup notation used previously (Sarkar and Guttman 2004; Hwang et al. 2005). A phylogeny constructed from nucleotide sequence alignments of the same 2,140 genes produced a similarly well-resolved tree with the same topology (not shown).

Based on the core genome, maximum pairwise values for *K*_A_ (the number of substitutions per nonsynonymous site) within phylogroups were calculated to be 0.018, 0.022, and 0.011 for phylogroups 1, 2, and 3, respectively. Between phylogroups, average estimates were 0.100, 0.094, and 0.067 for phylogroup 1 versus 2, 1 versus 3, and 2 versus 3, respectively. Maximum pairwise values for *K*_S_ (number of substitutions per synonymous site) within groups were 0.299, 0.312, and 0.124 for phylogroups 1, 2, and 3 respectively, whereas average *K*_S_ between groups were 1.584, 1.644, and 1.218 for phylogroup 1 versus 2, 1 versus 3, and 2 versus 3, respectively.

An analysis of consensus branching patterns within the set of individual core gene trees was performed to assess the level of support for the topology of the concatenated phylogeny at the individual gene level. An extended majority consensus tree is shown in supplementary figure S1, Supplementary Material online. Consensus support for phylogroups 1, 2, and 3 was 90%, 79%, and 86%, respectively, and overall, the consensus topology is in agreement with that of the core genome phylogeny at all partitions bar the relative placement of the two phylogroup 2 pathovars *cit7* and *syr6*; this discrepancy is reflected in the low bootstrap value (54%) at this node in the core genome tree. Two-thirds of gene trees support the clustering of phylogroups 2 and 3 but only 40% support the position of PG1 as a sister to these. This appears to be due to uncertainty about the placement of PG4 (represented by *oryz*); indeed, an earlier analysis based on four genes clustered PG4 with PG2 and PG3 (Hwang et al. 2005).

**Fig. 4.—:**
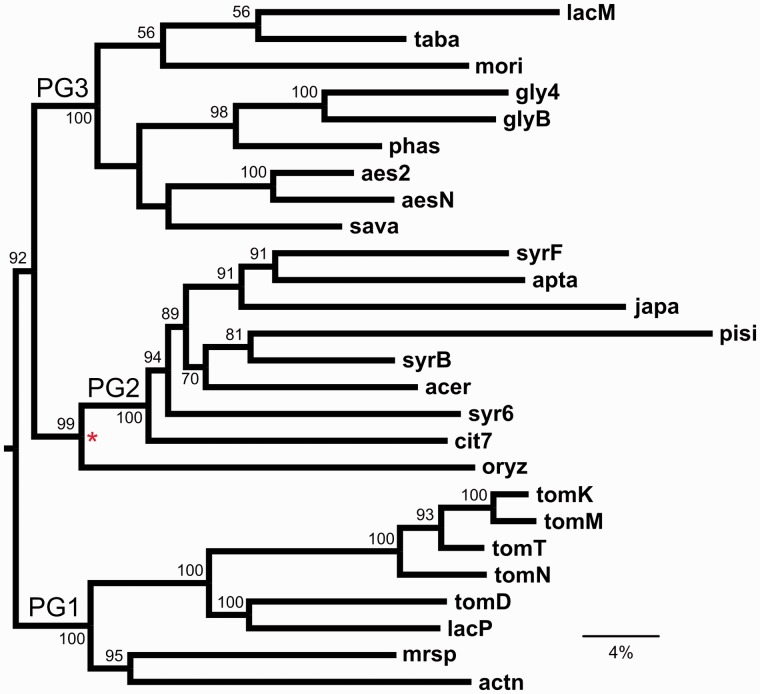
Genome content analysis. Numbers indicate percentage consensus support for that bifurcation from 1,000 bootstrap replicates. Phylogroups PG1, PG2, and PG3 are indicated. The red asterisk shows the only incongruence relative to the core genome phylogeny. Scale bar indicates 4% genomic dissimilarity, that is, percentage difference in terms of number of genes shared. Tree is rooted with *Pcan* (not shown).

Relationships among pathovars based on genome content similarities were also analyzed (fig. 4). Overall, there is a high degree of topological congruence between the genome content analysis and the core genome phylogeny. Phylogroups 1, 2, and 3 are reproduced monophyletically with 100% bootstrap support, while there is also substantial agreement in branching patterns within phylogroups. An exception is the placement of the PG4 strain *oryz*, which clusters with PG2 in the genome-content analysis (red asterisk). The major difference between the core genome and genome content phylogenies can be seen in the relative branch lengths with respect to phylogenetic depth within the two trees. Even after correction for multiple hits, the deeper branches in the genome-content analysis are very short, whereas the terminal branches are relatively extended.

### Gene Gain and Loss

**Fig. 5.—:**
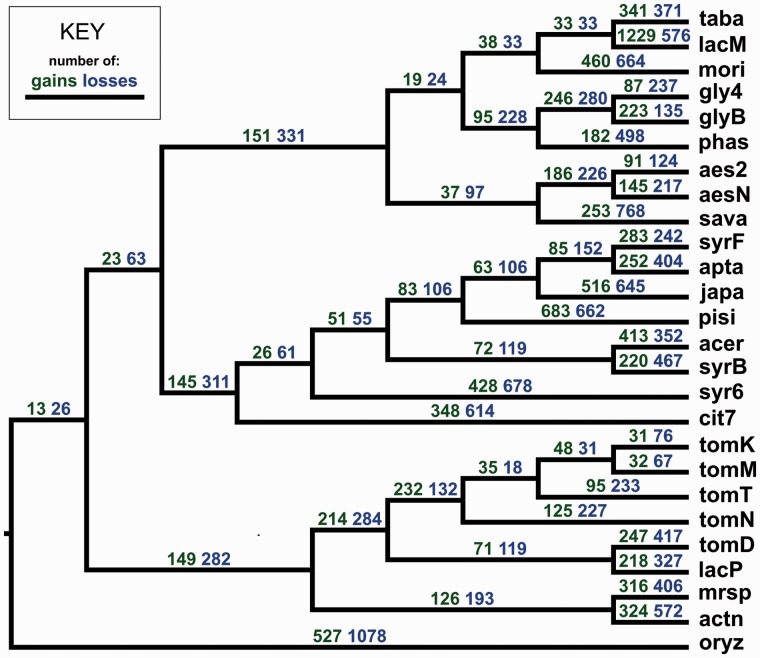
Gene gain and loss in *Pseudomonas syringae*. Inferred numbers of gains (green) and losses (blue) are given above each branch. Lineage-specific genes are included in gains along terminal branches. Values in all cases are rounded to the nearest whole number of events. Topology is based on figure 3; branch lengths are not to scale.

We used a stochastic mapping method to infer the number of gains and losses along all branches of the *P. syringae* core genome phylogeny (fig. 5). An underlying assumption of this method is that gain and loss events occur independently of one another. In reality, this is unlikely to be the case, because many genes may be gained (or lost) in a single transfer event (e.g., the acquisition of a plasmid). Thus, the results shown here likely overestimate the number of individual transfer events involved.

There is considerable variation in gene gain and loss among branches. Generally, values are higher along terminal branches and decrease along branches deeper in the phylogeny. The pathovar with the highest inferred number of terminal gains was *lacM* (1,229), followed by *pisi* (683) and *oryz* (527). On average, 6.4% of a pathovar’s total genome has been gained “recently” (i.e., subsequent to the divergence of a lineage from its nearest sister taxa), but this value increases to 13.2% when considering this proportion relative to the size of the flexible genome only. On terminal branches, lineages have lost 9.2% of the current genome size on average, and the three pathovars that have undergone the greatest number of losses are *oryz* (1,226), *sava* (768), and *syr6* (678).

High values of gain and loss for individual pathovars may be the result of either biologically relevant variation in genome content or an artifact of phylogenetic sampling. For example, *P. syringae* strains are known to harbor plasmids and the exceptionally large number of recent gains in *lacM* can be explained by the discovery of an approximately 1 Mb plasmid in this genome (Baltrus et al. 2011). Similarly, evidence for the presence of plasmids has been found for the *pisi* genome (Baltrus et al. 2011), although the exact size and gene content are not known. However, although *oryz* is thought to contain plasmids (Reinhardt et al. 2008), the high number of gains and losses seen in this pathovar is also due to the relatively long branch that separates it from other pathovars; *oryz* has had more time to accumulate differences in genome content.

**Fig. 6.—:**
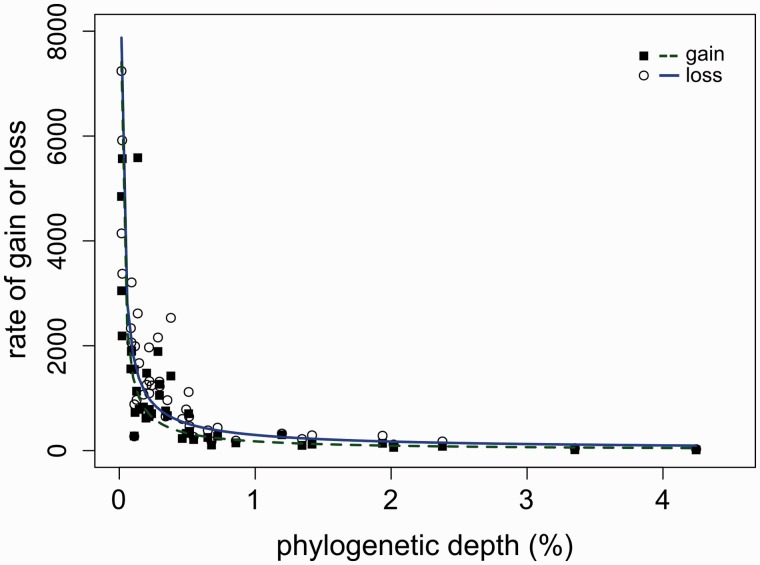
Rate of gene gain (filled squares) and loss (open circles) versus phylogenetic depth. Phylogenetic depth is calculated as the average distance (percent amino acid replacements per site) from the midpoint of each branch of the core genome phylogeny to its descendent tips; the *y* axis shows the branch-specific rate of gain or loss expressed as the number of inferred events per 1% amino acid divergence of the core genome phylogeny.

However, comparisons between absolute numbers of gains and losses per branch can be misleading, because these values are subject to the effects of phylogenetic sampling. To account for this, we calculated a rate of gain and loss relative to the amount of amino acid sequence divergence that has occurred along each branch of the core genome phylogeny (supplementary fig. S2, Supplementary Material online). To investigate the evolutionary dynamics of gain and loss at different depths of the phylogenetic tree, we plotted branch-specific rates against the phylogenetic depth of each branch, measured as the average distance from the midpoint of that branch to all descendent tips (fig. 6). The apparent rates of gain and loss decrease exponentially with respect to increasing phylogenetic depth, reflecting the much higher levels of observable gain and loss along branches closer to the tips of the tree (i.e., more recent in time).

### Source and Functions of Gained Genes

**Fig. 7.—:**
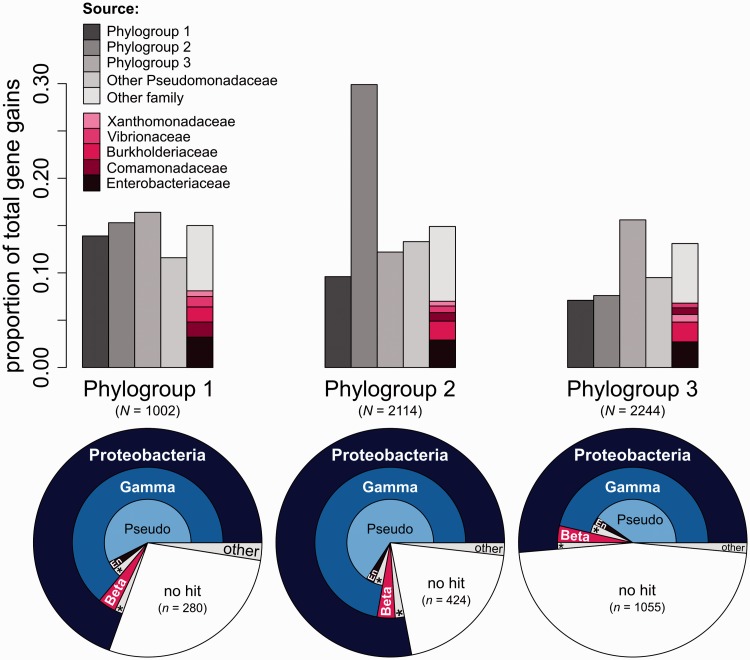
Sources of gained genes. Bar charts detail the fine-scale sources of genes gained recently in phylogroups 1, 2, and 3, as identified by best BLAST hit (*E* value ≤ 1 × 10^−20^). The *y* axis represents the proportion of total terminally acquired genes for each phylogroup. The category “other family” is further decomposed to show the family of the top five donors for each phylogroup (pink shaded boxes). Pie charts below show broader taxonomic classification of gained genes. Pseudo, Pseudomonadaceae; En, Enterobacteriaceae; asterisks denote “other” categories.

The extent of HGT within and between phylogroups was assessed using BLAST to determine the most similar sequence for all genes gained along terminal branches across the phylogeny (fig. 7). In phylogroups 2 and 3, the primary source of gained genes appears to have been other pathovars from within the same phylogroup. A significant contribution also comes from pathovars in other phylogroups, as well as from other *Pseudomonas* species outwith the *P. syringae* group. Taking phylogroups 1, 2, and 3 together, the ten most frequent putative donor genera were (in rank order) *Pseudomonas*, *Burkholderia*, *Vibrio*, *Escherichia*, *Xanthomonas*, *Ralstonia*, *Yersinia*, *Salmonella*, *Acidovorax*, and *Pectobacterium*. Transfers from these bacterial groups represent the great majority (∼86%) of the total number of recently transferred genes, and many contain species that are known to be associated with plant, water, or soil habitats. For example, both *Ralstonia* and *Xanthomonas*, themselves much-studied pathogens of other plant species (Mansfield et al. 2012), appear as frequent donors of genetic material into the genomes of phylogroup 2 and 3 pathovars. In addition, *Pectobacterium* contains phytopathogenic species that cause disease in both herbaceous and woody hosts (Toth et al. 2003), *Acidovorax* are pathogens of cucurbits (Schaad et al. 2008), whereas *Polaromonas* species are often associated with water and can be found in alpine meltwater (Jeon et al. 2004; Margesin et al. 2012), a known habitat of *P. syringae* (Morris et al. 2008). Species belonging to the Burkholderiaceae, from the *β*-proteobacteria, are among the top five putative donors from outwith the Pseudomonads for all three phylogroups, highlighting the potential contribution from relatively distant families.

Further analysis of putative donor species from other genera reveals the phylogenetic breadth of potential HGT sources (fig. 7). For all phylogroups, the majority of genes are donated from species within the *γ*-proteobacteria. A smaller proportion of genes are also acquired from more distant taxonomic groups such as the *α*- and *β*-proteobacteria and from other phyla such as the cyanobacteria. A significant fraction of genes in all phylogroups show “no hit,” indicating that many recently gained genes have not been previously sampled and are of unknown origin or function. The proportion of no hits in PG3 is approximately twice that relative to PG1 and PG2 due to the inflated number of genes specific to *P. syringae* pv. *lachrymans* str. M301315 (*lacM*), presumably encoded on the 1 Mb plasmid unique to this strain, the majority of which had no BLAST matches. The source of this large lineage-specific fragment of DNA remains to be elucidated.

Across all branches, the protein sequences of 6,378 genes gained with a probability of ≥0.8 were delimited into three basic categories based on OG: Functional protein, hypothetical protein, and phage-related protein (supplementary fig. S3*a*, Supplementary Material online). Across all branches, 59.6%, 37.8%, and 2.6% of gains were labeled as functional, hypothetical, or phage related, respectively. A further categorization of those sequences that were successfully annotated (i.e., functional proteins, as above) is provided in supplementary figure S3*b**,* Supplementary Material online.

Proteins inferred to be terminally acquired were queried versus a database of known T3SEs to ascertain the extent of recent transfer of this important family of genes. At the 1 × 10^−5^ BLAST threshold, this yielded 13, 34, and 36 hits for phylogroups 1, 2, and 3 respectively. However, applying the 80% identity over 80% query coverage homology rule narrowed this down to 8, 7, and 12 T3SEs for phylogroups 1, 2, and 3, respectively, accounting for a very small fraction (∼0.5%) of all recently gained genes. The terminal acquisition of T3SEs was not distributed evenly among pathovars. For example, within phylogroup 3, six were acquired along the terminal branch leading to *mori*, four along the branch leading to *lacM*, and two along the branch leading to *phas*. Only one T3SE protein, EGH71060.1 from *acer*, was both terminally acquired and strain specific.

## Discussion

We have investigated both the scale and dynamics of genome fluctuation in *P. syringae*. We demonstrate a history of extensive gene gain and loss that can rapidly alter the gene content of individual *P. syringae* lineages. Below, we discuss the wider implications of these results within the context of the role of HGT and gene loss in the differentiation and diversification of bacterial lineages, as well as the consequences of such fluctuation on the evolution of *P. syringae*. First, we consider some of the potential issues surrounding the use of whole-genome draft sequence data in comparative analyses that may have been previously underestimated in other studies.

### The Extent of Potential Errors in Draft Genomes

We applied a suite of quality control and data correction procedures to ensure the resultant patterns of orthology and gene presence/absence were as accurate as possible. The confounding effects of protein fragments, caused when a contig terminates in the middle of a CDS, were of particular significance. Our observations emphasize the care required when drawing inferences from uncorrected patterns of orthology constructed using multiple (often hundreds of) contig draft genome sequences in conjunction with popular orthology methods such as OrthoMCL.

Not accounting for this source of error can introduce considerable bias in a number of common comparative genomic analyses. For example, we estimated that approximately 77% of inferred lineage-specific proteins and approximately 13% of the precorrected number of OGs could be apportioned to error. Even fundamental descriptive metrics for a genome, such as the number of proteins it encodes, can be substantially overestimated by the presence of protein fragments. For example, the combined data correction procedures applied here reduce the number of proteins encoded by the *P. syringae* pv. *pisi* genome from 9,160 to 5,085, a reduction of approximately 44% (i.e., almost one-half of the original number). The mean reduction in proteome size across the 27 genomes in this study is approximately 17%. These observations simultaneously reveal the possible level of error in the annotation of genome sequences and the potential for the use of orthology to reduce the problem.

Errors in the observed patterns of orthology among proteins can adversely affect the estimation of core- and pan-genome sizes and can lead to the miscalculation of the level of paralogy and gene duplication within genomes. For example, the core genome of *P. syringae* was recently estimated at 1,856 genes, approximately 740 genes smaller than that reported here (O’Brien et al. 2012). Problems caused by protein fragments may be one reason for this discrepancy. In addition, a recent study on genome fluctuation in cyanobacteria suggests that a large number of paralogs within the genomes of certain groups of cyanobacteria may be indicative of a higher rate of gene duplication (Larsson et al. 2011). However, it is also possible that a large fraction of the apparent paralogs may be attributable to the presence of protein fragments.

### Phylogenetics of the Core and Flexible Genomes

We inferred the evolutionary relationships among pathovars based on both the evolution of the core genome and gene repertoire similarity of the flexible genome. Genome content similarity has been predicted to be a poor indicator of phylogenetic relatedness, where the rapidity of gene turnover is expected to blur inherited patterns of gene presence and absence (Hao and Golding 2006; Vernikos et al. 2007; Touchon et al. 2009). Thus, topological discordance between phylogenies based on these two genome components is thought to be an indicator of extensive HGT among lineages (Shapiro et al. 2012). However, the congruence observed between the *P. syringae* core genome and genome content phylogenies suggests that, despite high rates of HGT and gene loss, an accurate and robust phylogenetic signal is recoverable from patterns of presence and absence among genes of the flexible genome.

Varying degrees of topological congruence between the core and flexible components of bacterial genomes have been reported elsewhere. For example, an investigation of genome evolution in *Prochlorococcus* (Kettler et al. 2007) shows near perfect congruence, whereas studies of other bacteria, for example, *Vibrio* (Shapiro et al. 2012) and *Rickettsia* (Georgiades et al. 2011), show near total discordance. Reasons for this variation may include differences in the rate and extent of genome fluctuation experienced by these groups, the level of divergence among lineages, or perhaps ecological factors associated with these different species. However, both *Prochlorococcus* and *Vibrio* are ocean-dwelling species where the opportunity for HGT is presumably relatively high, yet they show opposite patterns. Moreover, *Rickettsia* species are obligate intracellular parasites of eukaryotic hosts with presumably relatively limited opportunity for gene gain. However, *Rickettsia* genomes are known to be shrinking in size (Georgiades et al. 2011), and so it may be the case that the genome content topology is driven by large-scale differential gene loss as these species adapt to different hosts. Patterns of divergence also fail to correlate with the level of congruence. These groups of bacteria are generally highly similar, having 16S ribosomal RNA nucleotide identities ranging between approximately 97% (*Prochlorococcus*) and 100% (*Vibrio*). Contrary to what might be expected, the group with the greatest 16S divergence (*Prochlorococcus*) shows the most congruence between phylogenies despite presumably having more time to accumulate differences in genome content. Furthermore, *P. syringae* and *Rickettsia* differ dramatically in the level of congruence despite having similar 16S identities (both ∼99%). In all cases, the lack of signal seems not to be the issue, because all groups show considerable variation in genome repertoires among lineages (Kettler et al. 2007; Georgiades et al. 2011; Shapiro et al. 2012). Certainly, *P. syringae* provides a clear example that a high level of genome fluctuation does not preclude a high degree of congruence between trees derived from the core genome and genome content comparisons.

### Species Definition in *P. syringae*

On the basis of over 1 million orthologous nucleotide sites of the core genome, we estimate that the three *P. syringae* phylogroups are as diverged from each other as other taxa classified as separate species or even genera. For example, *K*_A_ between the sister clade phylogroups 2 and 3 is estimated to be 0.06 substitutions per site. This value is greater than that for a number of other species comparisons, including *Escherichia coli–Salmonella enterica* and *Neisseria gonorrhoeae*–*N. polysaccharea* (Ochman et al. 1999). *K*_A_ divergence for comparisons between phylogroups 1 and 2/3 are greater yet and are comparable with *K*_A_ between two other “bona fide” *Pseudomonas* species, *P. aeruginosa* and *P. putida* (see fig. 2 of Ochman et al. 1999). Values of *K*_S_ between phylogroups are all >1, suggesting that substitution at synonymous sites is approaching saturation. Although high *K*_S_ values are unreliable as point estimates, species pairs in a number of other genera (including *Buchnera*, *Mycobacterium*, *Bacillus*, and *Pseudomonas*) all have lower (<1) estimates of *K*_S_ (Ochman et al. 1999). In addition, consensus analysis from the set of individual core gene trees shows that a high percentage of core genes support the delineation of these lineages into three major groups (90%, 79%, and 86% for phylogroups 1, 2, and 3, respectively), suggesting that the recombination of core alleles between phylogroups does not occur at a level sufficient to erode the boundaries between groups. As such, and notwithstanding the ongoing debate as to the validity of such terms (Lawrence 2002; Doolittle and Papke 2006; Caro-Quintero and Konstantinidis 2011; Hanage 2013), phylogroups may be effectively described as nascent species.

There has been ongoing debate regarding the delimitation of the *P. syringae* complex into distinct species (Gardan et al. 1992; Sarkar and Guttman 2004; Bull et al. 2010). Sarkar and Guttman (2004) suggest that due to the ecological similarity between pathovars across phylogroups, the sharing of core alleles among phylogroups, and evidence of extensive HGT between phylogroups, *P. syringae* should remain as a single species. However, the results presented here suggest that these factors may not necessarily hinder speciation, because neither is sufficient to halt the accumulation of divergence from either the core or the flexible components of the *P. syringae* pan genome. We suggest that the data presented in this study support the delineation of *P. syringae* into a number of distinct species based on the phylogroup partitioning that is already well established. The strains *gly4*, *glyB*, and *sava* within phylogroup 3 have been previously referred to as strains of *P. savastanoi* (Bull et al. 2010; Rodríguez-Palenzuela et al. 2010; Qi et al. 2011; Ramos et al. 2012); clearly, the other pathovars within phylogroup 3 (*taba*, *lacM*, *mori*, *phas*, *aes2,* and *aesN*) should be classified in the same species (also see Bull et al. 2010, who state that phylogroup 3 strains should be classified as *P. amygdali* if unification occurs, because *P. savastanoi* and others are later synonyms). The type strain for *P. syringae*, NCPPB 281, clusters in phylogroup 2 along with the complete genome *P. syringae* pv. *syringae* str. B728a (*syrB*) (Parkinson et al. 2010), whereas it appears that species names for phylogroups 1 and 4 have not yet been proposed.

### Genome Repertoire Dynamics and Niche Adaptation in *P. syringae*

The quantification of gene gain and loss suggests that both have been extensive in the evolutionary history of the *P. syringae* species complex and that the genomes of these pathovars may undergo considerable fluctuation even over relatively short evolutionary time periods. A large fraction of the total gain and loss was observed along terminal branches leading to individual pathovars, in agreement with previous studies investigating genome dynamics in other bacterial species (Hao and Golding 2006; Kettler et al. 2007; Lefébure and Stanhope 2007; Touchon et al. 2009).

When absolute numbers of gene gains and losses are compared with the rate of sequence divergence in proteins encoded by the core genome, the results are striking. At shallower phylogenetic depths (representative of terminal branches), we estimate that hundreds or thousands of genes may be gained (and lost) in the same time period as a 1% amino acid divergence. For example, the terminal branch leading to the complete genome of *P. syringae* pv. *phaseolicola* str. 1448A (*phas*) has gained 182 genes over a divergence of about 0.25% (equivalent to 728 genes over 1% divergence). Estimates of rates of gene loss are of at least the same magnitude. However, across the *P. syringae* complex as a whole, more than 50% of the genome remains as core, despite divergence values up to nearly 10%. This suggests, in agreement with other studies, that the majority of gained genes are transient and are quickly discarded from recipient genomes (Tettelin et al. 2005; Hao and Golding 2006; Kuo and Ochman 2009; Touchon et al. 2009). This leads to a perspective bias, where the rates of gene gain and loss both appear to decrease with increasing phylogenetic depth, because over longer time periods only a fraction of older gains have persisted and remain observable (Rocha 2008).

Based on seven closely related genomes of the *Bacillus cereus* group with divergences of the order of approximately 1% nucleotide substitutions per site, Hao and Golding (2006) estimated the recent rate of turnover to be such that a gene may be gained and lost approximately 5 times during the time required to observe a single-nucleotide substitution per site and conclude that the rate of transfer is at least comparable with that of nucleotide replacement. It is clear that the estimated rate of gene turnover relative to sequence divergence is highly dependent on the phylogenetic depth over which it has been calculated (fig. 6). However, using a core genome tree based on nucleotide sequences, we calculate the rate for *P. syringae* terminal branches that are equivalent in length to that of the *B. cereus* group to be perhaps four orders of magnitude greater. This highlights the predominance of HGT as an adaptive force for *P. syringae*, as well as the potential scale of the difference in rates of HGT between different bacterial groups.

We also observe an excess of gene losses relative to gene gains for the majority of branches of the core genome phylogeny. Taken at face value, this suggests a recent contraction of the *P. syringae* genome. However, given that extant strains of *P. syringae* have above-average sized genomes compared with other *γ*-proteobacteria, it seems unlikely that they have undergone prolonged genome reduction. It has also been demonstrated that methodological biases or data issues can overestimate the rate of either gain or loss (Zhaxybayeva et al. 2007; Hao and Golding 2008).

Generally, the most frequent donors of genetic material appear to have been lineages within the same phylogroup as the recipient genome. Smaller contributions have also come from the other phylogroups and from other Pseudomonads. This might be expected because the rate of homologous recombination decreases exponentially with sequence divergence (Majewski 2001), reducing the incidence of HGT when genes are carried into a new genome via the homologous recombination of flanking regions (Touchon et al. 2009; Polz et al. 2013). Genes arriving from outwith the *Pseudomonas* genus have been acquired from a diverse range of donor species, many of which are known to be soil inhabiting, plant associated, or present in water systems. GO analysis shows that gained genes are drawn from a diverse range of functional categories and are therefore likely to contribute a correspondingly diverse range of novel phenotypes to recipient lineages. *Pseudomonas syringae* is well known for its ability to exist in a multitude of both plant and environmental niches, and the results presented here suggest that such ecological versatility is facilitated by high rates of gene gain into the flexible genome.

These results suggest an underlying structure to gene gain in *P. syringae* that is based on both phylogeny and ecology. An attractive extrapolation of such structuring of HGT is the hypothesis of “niche-specific gene pools” that contribute to adaptation within an ecologically determined framework, via the transfer of genes that are selectively advantageous within a certain niche (Popa et al. 2011; Smillie et al. 2011; Polz et al. 2013). In the case of *P. syringae*, this framework may be determined by the ecology and population structure of the numerous plant host species on which these pathovars thrive. In this way, the expansion of *P. syringae* lineages onto a new host species may occur when newly acquired genes from other species that already inhabit that environment prove to be selectively advantageous to the recipient lineage, thus facilitating a host jump.

### Divergence via Differential Adaptation in *P. syringae*

As the extent and prevalence of HGT has become increasingly evident, much discussion has focused on the implications of HGT for the phylogenetic reconstruction of prokaryotic lineages and the validity of the species definition. More recently, however, models have emerged in which HGT can be an active facilitator, rather than a restrictor, of the evolution of population structure, ecological differentiation, and speciation (Ochman et al. 2005; Shapiro et al. 2012; Polz et al. 2013). These ideas rely on the understanding that although rates of HGT and gene loss can be very high, the underlying dynamics of this fluctuation are highly structured in such a way that they actively promote the differentiation of subpopulations of bacteria into distinct genotypic clusters.

HGT and gene loss can directly facilitate the accumulation of divergence between bacterial populations via the differential adaptation of lineages into distinct ecological niches (Ochman et al. 2005; Polz et al. 2013). This mechanism may be operating in the evolution of *P. syringae* and may explain the broad host range of the *P. syringae* species complex while maintaining the host specificity of individual pathovars. *Pseudomonas syringae* genomes show a turbulent history of genome fluctuation, with evidence of high levels of gene gain and loss in all lineages across the species complex. Gained genes are functionally diverse and appear to be gained from sources that are both phylogenetically and ecologically structured. The degree of host specificity shown by many individual pathovars suggests that their gene repertoires are specifically adapted to a given niche environment (Morris et al. 2000; Hunter and Taylor 2006; Sarkar et al. 2006). It is likely that this genomic “tailoring” is achieved through the continuous uptake of a large number of genes via HGT, but the retention of only those few niche-specific genes that confer an advantage in a given host environment.

In *P. syringae*, much attention has been focused on the contribution of effector genes of the type III secretion system (T3SS) toward adaptation and pathogenicity. Although the distribution of the approximately 57 currently described effector families across the *P. syringae* species complex has been well characterized, the relationship between the type 3 secretion effector (T3SE) repertoire and adaptation is complex and remains to be fully elucidated (Sawada et al. 1999; Ma et al. 2006; Lindeberg et al. 2009, 2012; Baltrus et al. 2011; O'Brien et al. 2012). For example, strains that are pathogenic on the same host species can have divergent effector complements (Almeida et al. 2009; Baltrus et al. 2011; O’Brien et al. 2011), suggesting that *P. syringae* lineages are able to adapt to a given environment using multiple variations of effector repertoires (Lindeberg et al. 2012). The acquisition of novel T3SEs may be indicative of adaptive transitions into new niche environments (Sarkar et al. 2006). If T3SEs were solely responsible for the adaptive diversification of *P. syringae* pathovars, it might be expected to find a unique repertoire of T3SEs for each transition onto a different host species and that recent host jumps correspond to recent gains of novel T3SE genes. However, we find that not all pathovars have recently gained T3SE genes and that of those strains that show evidence of recent T3SE gain, only one was also specific to that lineage. Taken together, these observations suggest that host adaptation and the evolution of host specificity may be facilitated by the acquisition of genes that are not directly involved in the T3SS or by molecular adaptations in virulence genes that are already present within the genome.

When acquired genes confer the ability of that lineage to occupy a new ecological niche, such as a new host species, a novel disease can be the result. In the case of *P. syringae*, a number of newly emergent diseases have been reported in the last decade, including bleeding canker of horse chestnut, caused by *P. syringae* pv. *aesculi* (Webber et al. 2008), and bacterial canker of kiwi caused by pv. *actinidiae* (Balestra et al. 2010). Both of these pathovars have recently acquired genes involved in the catabolism of plant-derived aromatic compounds such as derivatives of lignin that are likely to be central in their ability to inhabit (and cause disease in) the woody parts of their respective host species (Green et al. 2010; Steele et al. 2010; Marcelletti et al. 2011). *Pseudomonas syringae* pv. *aesculi* has recently become epidemic across much of north-west Europe, and the success of this pathogen appears to be due, at least in part, to the ability of this species to acquire genes from disparate sources that facilitate adaptation into distinct ecological niches.

## Supplementary Material

Supplementary figures S1–S3 and table S1 are available at *Genome Biology and Evolution* online (http://www.gbe.oxfordjournals.org/).

Supplementary Data
