# Supplementary material for: The Extent of Genome Flux and Its Role in the Differentiation of Bacterial Lineages
Source: Genome Biol Evol. 2014 Jun 12;6(6):1514–29. doi: 10.1093/gbe/evu123 (PMC4079204; doi:10.1093/gbe/evu123)
Supplement: Supplementary Data [file supp_6_6_1514__index.html]

The extent of genome flux and its role in the differentiation of bacterial lineages — The Extent of Genome Flux and Its Role in the Differentiation of Bacterial Lineages — Supplementary Data 

# The Extent of Genome Flux and Its Role in the Differentiation of Bacterial Lineages

## Supplementary Data

files

**Files in this Data Supplement:**

- Supplementary Data - pdf file
