# Supplementary material for: The Extent of Genome Flux and Its Role in the Differentiation of Bacterial Lineages
Source: Genome Biol Evol. 2014 Jun 12;6(6):1514–29. doi: 10.1093/gbe/evu123 (PMC4079204; doi:10.1093/gbe/evu123)
Supplement: Supplementary Data [file supp_evu123_SupplementaryMaterial_GBE.pdf]

Table S1.—Complete information for 27 *P. syringae* pathovars used in this study.

Pathovar name	Strain	Tag*	Host species	Genome size (Mb)	*n* proteins†	Genome status (*n* contigs)	Reference
aceris	M302273PT	acer	*Acer* Sp. (maple), USA	6.183	6185	Draft (1179)	Baltrus et al. (2011)
actinidiae	M302091	actn	*Actinidia delicosa* (kiwi fruit), Japan	5.849	5626	Draft (941)	Baltrus et al. (2011)
aesculi	2250	aes2	*Aesculus hippocastanum* (European horse chestnut), UK	5.926	5619	Draft (776)	Green et al. (2010)
aesculi	NCPPB 3681	aesN	*Aesculus indica* (Indian horse chestnut), India	5.836	5649	Draft (841)	Green et al. (2010)
aptata	DSM 50252	apta	*Beta vulgaris* (sugar beet), USA	6.243	6368	Draft (3776)	Baltrus et al. (2011)
'*cit7*'	Cit7	cit7	*Citrus sinensis* (navel orange), unknown	6.075	7145	Draft (2655)	Baltrus et al. (2011)
glycinea	B076	glyB	*Glycine max* (soybean), USA	6.236	5580	Draft (104)	Qi et al. (2011)
glycinea	race 4	gly4	Not explicitly stated	5.905	5207	Draft (108)	Qi et al. (2011)
japonica	M301072PT	japa	*Hordeum vulgare* (barley), Japan	6.381	8796	Draft (4661)	Baltrus et al. (2011)
lachrymans	M301315	lacM	*Cucumis sativus* (cucumber), Japan	7.727	6691	Draft (791)	Baltrus et al. (2011)
lachrymans	M302287PT	lacP	*Cucumis sativus* (cucumber), USA	5.895	5636	Draft (798)	Baltrus et al. (2011)
maculicola	ES4326	Pcan‡	*Raphanus sativus* (radish), unknown	6.221	6101	Draft (878)	Baltrus et al. (2011)
mori	M301020	mori	*Morus alba* (white mulberry), Japan	6.393	7544	Draft (3414)	Baltrus et al. (2011)
morsprunorum	M302280PT	mrsp	*Prunus domesticus* (European plum), unknown	6.039	5837	Draft (969)	Baltrus et al. (2011)
oryzae	1_6	oryz	*Oryza sativa* (rice plant), unknown	6.704	7837	Draft (2855)	Reinhardt et al. (2008)
phaseolicola	1448A	phas	*Phaseolus vulgaris* (common bean), Ethiopia	6.112	5172	Complete (1 chr., 2 plasmids)	Joardar et al. (2005)
pisi	1704B	pisi	*Pisum sativum* (pea), France	6.521	9160	Draft (5099)	Baltrus et al. (2011)
savastanoi	NCPPB 3335	sava	*Olea europaea* (olive tree), France	5.759	4898	Draft (403)	Rodriguez-Palenzuela et al. (2010)
syringae	B728a	syrB	Snap bean leaflet, unknown	6.093	5089	Complete (1 chr.)	Feil et al. (2005)
syringae	FF5	syrF	*Pyrus calleryana* (ornamental pear), USA	5.659	8445	Draft (4578)	Sohn et al. (2012)
syringae	642	syr6	Isolated from a "small newly emerging weedy plant" (Clarke *et al.*, 2010), USA	5.809	5274	Draft (296)	Clarke et al. (2010)
tabaci	ATCC 11528	taba	*Nicotiana tabacum* (tobacco plant), unknown	6.068	6467	Draft (1405)	Studholme et al. (2009)
tomato	DC3000	tomD	*Solanum lycopersicum* (tomato), Guernsey (Channel Is., UK)	6.538	5619	Complete (1 chr., 2 plasmids)	Buell et al. (2003)
tomato	K40	tomK	*Solanum lycopersicum* (tomato), unknown	6.154	5853	Draft (582)	Cai et al. (2011)
tomato	Max13	tomM	*Solanum lycopersicum* (tomato), France	6.105	5749	Draft (349)	Vinatzer et al. (unpublished)
tomato	NCPPB 1108	tomN	*Solanum lycopersicum* (tomato), Jersey (Channel Is., UK)	6.082	5619	Draft (304)	Cai et al. (2011)
tomato	T1	tomT	*Solanum lycopersicum* (tomato), Canada	6.146	5702	Draft (122)	Almeida et al. (2009)

*4-letter identifier used in this study.

†Based on the number of published protein sequences available at NCBI.

‡The pathovar *maculicola* has been recently found to have been originally misidentified, and should in fact be renamed *Pseudomonas cannabina* pv. *alisalensis* (reported in Baltrus et al. 2011); thus the tag '*Pcan'* has been used in this study.

Literature Cited

Almeida NF, et al. 2009. A draft genome sequence of *Pseudomonas syringae* pv. *tomato* T1 reveals a type III effector repertoire significantly divergent from that of *Pseudomonas syringae* pv. *tomato* DC3000. Mol Plant Microbe Interact. 22:52–62.

Baltrus DA, et al. 2011. Dynamic evolution of pathogenicity revealed by sequencing and comparative genomics of 19 *Pseudomonas syringae* isolates. PLoS Pathog. 7:e1002132.

Buell CR, et al. 2003. The complete genome sequence of the *Arabidopsis* and tomato pathogen *Pseudomonas syringae* pv. *tomato* DC3000. Proc Natl Acad Sci USA. 100:10181–10186.

Cai R, et al. 2011. The plant pathogen *Pseudomonas syringae* pv. *tomato* is genetically monomorphic and under strong selection to evade tomato immunity. PLoS Pathog. 7:e1002130.

Clarke CR, Cai R, Studholme DJ, Guttman DS, Vinatzer BA. 2010. *Pseudomonas syringae* strains naturally lacking the classical *P. syringae* hrp/hrc locus are common leaf colonizers equipped with an atypical type III secretion system. Mol Plant Microbe Interact. 23:198–210.

Feil H, et al. 2005. Comparison of the complete genome sequences of *Pseudomonas syringae* pv. *syringae* B728a and pv. *tomato* DC3000. Proc Natl Acad Sci USA. 102:11064–11069.

Green S, et al. 2010. Comparative genome analysis provides insights into the evolution and adaptation of *Pseudomonas syringae* pv. *aesculi* on *Aesculus hippocastanum*. PLoS ONE. 5:e10224.

Joardar V, et al. 2005. Whole-genome sequence analysis of *Pseudomonas syringae* pv. *phaseolicola* 1448A reveals divergence among pathovars in genes involved in virulence and transposition. J Bacteriol. 187:6488.

Qi M, Wang D, Bradley CA, Zhao Y. 2011. Genome sequence analyses of *Pseudomonas savastanoi* pv. *glycinea* and subtractive hybridization-based comparative genomics with nine Pseudomonads. PLoS ONE. 6:e16451.

Reinhardt JA, et al. 2008. De novo assembly using low-coverage short read sequence data from the rice pathogen *Pseudomonas syringae* pv. *oryzae*. Genome Res. 19:294–305.

Rodríguez-Palenzuela P, et al. 2010. Annotation and overview of the *Pseudomonas savastanoi* pv. *savastanoi* NCPPB 3335 draft genome

reveals the virulence gene complement of a tumour-inducing pathogen of woody hosts. Environ Microbiol. 12:1604–1620.

Sohn KH, Jones JDG, Studholme DJ. 2012. Draft genome sequence of *Pseudomonas syringae* pathovar *syringae* strain FF5, causal agent of stem tip dieback disease on ornamental pear. J Bacteriol. 194:3733–3734.

Studholme DJ, et al. 2009. A draft genome sequence and functional screen reveals the repertoire of type III secreted proteins of *Pseudomonas syringae* pathovar *tabaci* 11528. BMC Genomics. 10:395. doi: 10.1186/1471-2164-10-395.

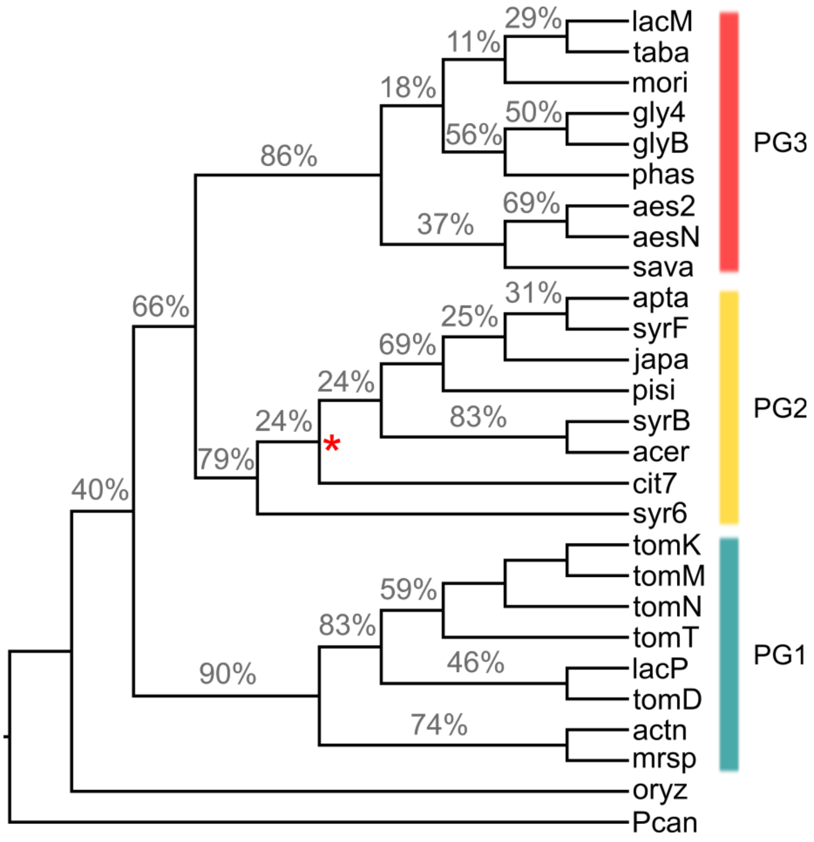

SUPPLEMENTARY FIG. S1.— Extended majority consensus tree. Grey numbers indicate the consensus support for a given branch, given as the proportion of times (expressed as a percentage) a particular branch splits the set of taxa into two subsets separated by that branch. The asterisk indicates the only incongruence in branching pattern relative to the core-genome phylogeny.

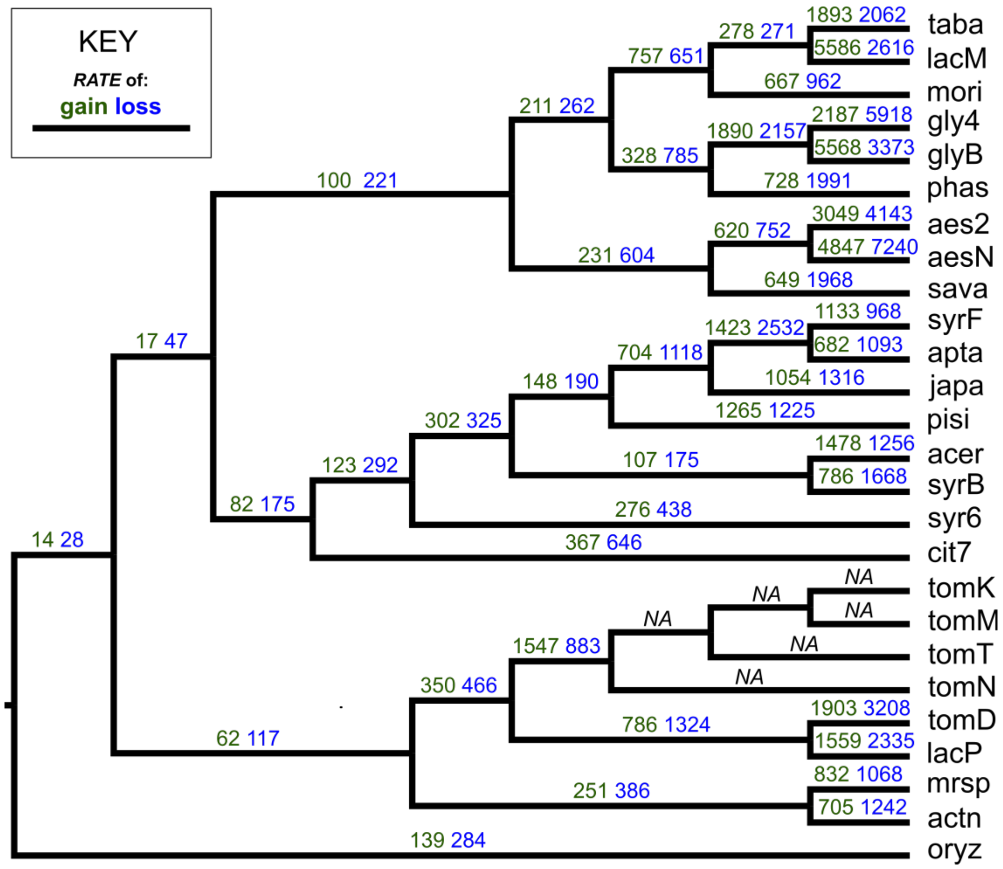

Supplementary Fig. S2.— Rate of gain and loss. Numbers above each branch indicate the ratio of the number events along a given branch to the length of that branch, i.e. the number of gains (green) or losses (blue) per percentage amino-acid divergence of the core-genome phylogeny. These values are not given for some branches with zero branch length (*NA*). Lineage-specific genes are included in gains along terminal branches. Topology is based on the core-genome phylogeny and branch lengths are not to scale.

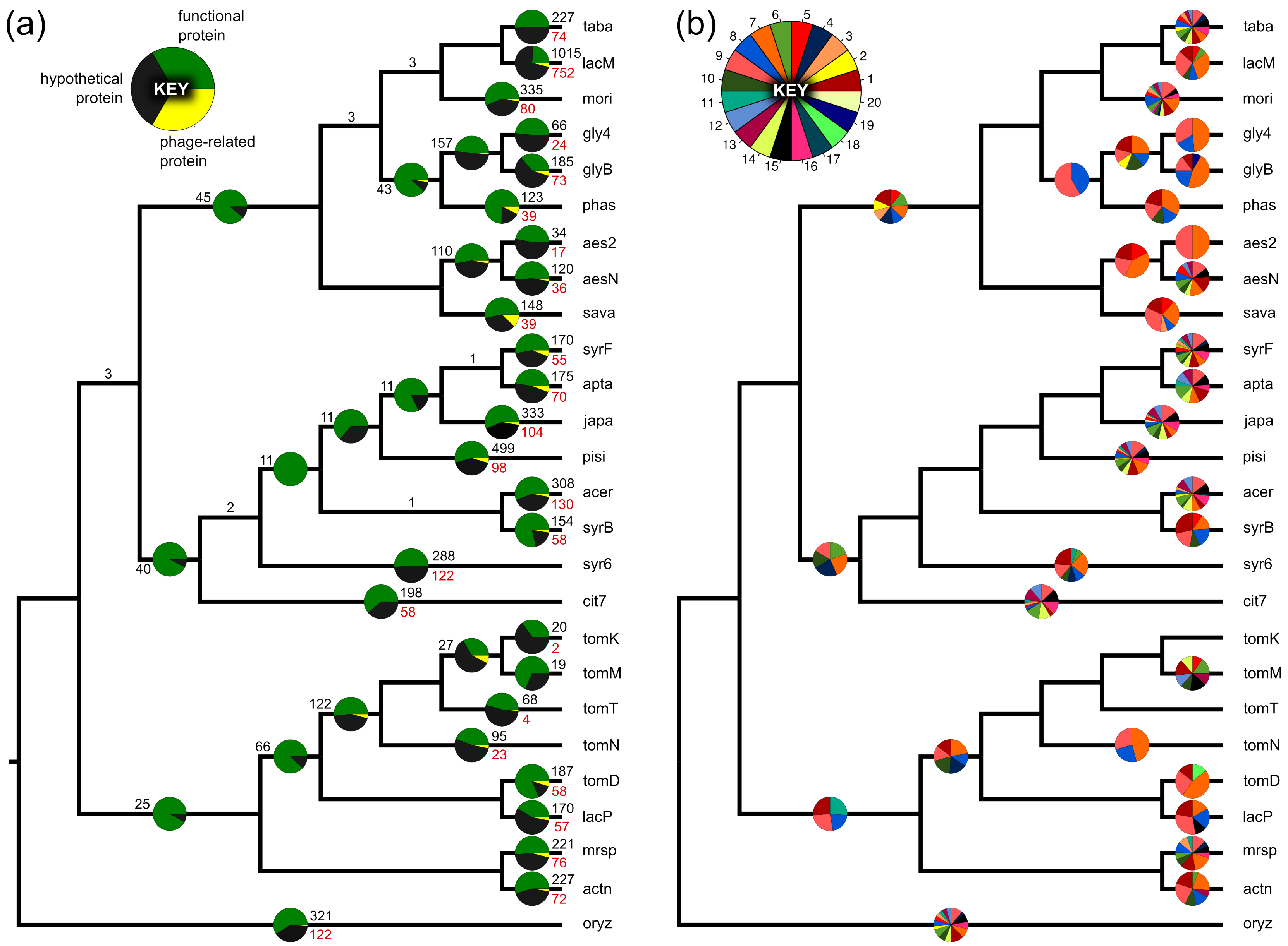
(a)
functional protein
hypothetical protein
KEY
phage-related protein
taba
lacM
mori
gly4
glyB
phas
aes2
aesN
sava
syrF
apta
japa
pisi
acer
syrB
syr6
cit7
tomK
tomM
tomT
tomN
tomD
lacP
mrsp
actn
oryz
(b)
KEY
taba
lacM
mori
gly4
glyB
phas
aes2
aesN
sava
syrF
apta
japa
pisi
acer
syrB
syr6
cit7
tomK
tomM
tomT
tomN
tomD
lacP
mrsp
actn
oryz

Supplementary Fig. S3.—Functional categorisation of gained genes. (a) Simple categorisation. Numbers above each branch indicate the total number of gains (≥ 0.8) for that branch; red numbers on terminal branches indicate the number of terminal gains that are lineage-specific, *i.e.* unique to the given lineage. Pies along each branch represent the classification of genes into one of three basic categories: 'functional protein' (green), 'hypothetical protein' (black) or 'phage-related protein' (yellow). Only branches with ≥ 10 gains are delimited into pies. The topology of the cladogram is based on the core-genome phylogeny (branch lengths not to scale). (b) Gene ontology categorisation (molecular function, GO level 3). Genes from (a) in the 'functional protein' category are further decomposed into functional groups based on gene ontology (GO) information. Only GO terms with ≥ 3 sequences are shown. Functional descriptions corresponding to the numbers in the key are given in the table below (for further detail regarding GO terms see www.geneontology.org).

Table of GO categories:

#	Description	#	Description
**1**	Transferase activity	**11**	Transmembrane transporter activity
**2**	Lysase activity	**12**	Amine binding
**3**	Signal transducer activity	**13**	Carboxylic acid binding
**4**	Sequence-specific DNA binding transcription factor activity	**14**	Vitamin binding
**5**	Ion binding	**15**	Cofactor binding
**6**	Substrate-specific transporter activity	**16**	Ligase activity
**7**	Nucleic acid binding	**17**	Isomerase activity
**8**	Nucleotide binding	**18**	Recombinase activity
**9**	Hydrolase activity	**19**	Protein binding
**10**	Oxidoreductase activity	**20**	Metal cluster binding
